# 2-De­oxy-2,3-*O*-isopropyl­idene-2,4-di-*C*-methyl-β-l-arabinose

**DOI:** 10.1107/S1600536809005777

**Published:** 2009-02-21

**Authors:** K. Victoria Booth, Sarah F. Jenkinson, George W. J. Fleet, David J. Watkin

**Affiliations:** a Department of Organic Chemistry, Chemistry Research Laboratory, Department of Chemistry, University of Oxford, Oxford OX1 3TA, England; b Department of Chemical Crystallography, Chemistry Research Laboratory, Department of Chemistry, University of Oxford, Oxford OX1 3TA, England

## Abstract

X-ray crystallography unequivocally confirmed the stereochemistry of the C atom at position 2 in the carbon scaffold of the title mol­ecule, C_10_H_18_O_4_. The pyran­ose ring exists in a chair conformation with the methyl group on the C atom in the 2 position in an equatorial configuration. The absolute stereochemistry was determined from the starting material. The crystal structure consists of O—H⋯O hydrogen-bonded chains of mol­ecules running parallel to the *b* axis.

## Related literature

For de­oxy sugars see: Becker & Lowe (2003 ▶); Yoshihara *et al.* (2008 ▶); Gullapalli *et al.* (2007 ▶). For a related structure see: Booth *et al.* (2007 ▶).
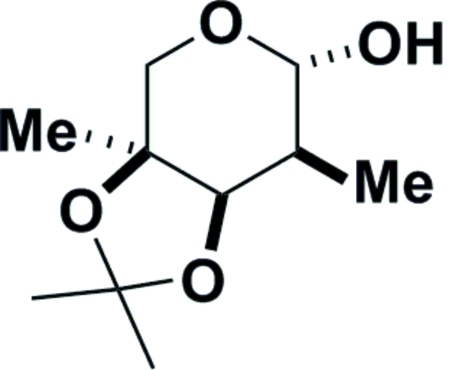


## Experimental

### 

#### Crystal data

C_10_H_18_O_4_
*M* *_r_* = 202.25
Monoclinic, 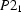

*a* = 6.0641 (3) Å
*b* = 13.4016 (7) Å
*c* = 6.8287 (3) Å
β = 102.596 (2)°
*V* = 541.60 (5) Å^3^
*Z* = 2
Mo *K*α radiation
μ = 0.10 mm^−1^
*T* = 150 K
0.50 × 0.20 × 0.20 mm

#### Data collection

Nonius KappaCCD diffractometer
Absorption correction: multi-scan (*DENZO*/*SCALEPACK*; Otwinowski & Minor, 1997 ▶) *T*_min_ = 0.89, *T*_max_ = 0.98
5025 measured reflections
1266 independent reflections
1183 reflections with *I* > 2σ(*I*)
*R*_int_ = 0.036

#### Refinement

*R*[*F* ^2^ > 2σ(*F* ^2^)] = 0.030
*wR*(*F* ^2^) = 0.073
*S* = 0.98
1266 reflections
127 parameters
1 restraint
H-atom parameters constrained
Δρ_max_ = 0.16 e Å^−3^
Δρ_min_ = −0.16 e Å^−3^

### 

Data collection: *COLLECT* (Nonius, 2001 ▶).; cell refinement: *DENZO*/*SCALEPACK* (Otwinowski & Minor, 1997 ▶); data reduction: *DENZO*/*SCALEPACK*; program(s) used to solve structure: *SIR92* (Altomare *et al.*, 1994 ▶); program(s) used to refine structure: *CRYSTALS* (Betteridge *et al.*, 2003 ▶); molecular graphics: *CAMERON* (Watkin *et al.*, 1996 ▶); software used to prepare material for publication: *CRYSTALS*.

## Supplementary Material

Crystal structure: contains datablocks global, I. DOI: 10.1107/S1600536809005777/lh2774sup1.cif

Structure factors: contains datablocks I. DOI: 10.1107/S1600536809005777/lh2774Isup2.hkl

Additional supplementary materials:  crystallographic information; 3D view; checkCIF report

## Figures and Tables

**Table 1:** Hydrogen-bond geometry (Å, °)

*D*—H⋯*A*	*D*—H	H⋯*A*	*D*⋯*A*	*D*—H⋯*A*
O12—H121⋯O1^i^	0.86	1.93	2.786 (3)	179

Symmetry code: (i) 
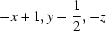
.

**Fig. 1.**
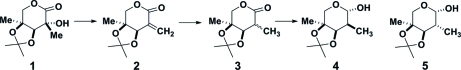
Synthetic Scheme

**Fig. 2.**
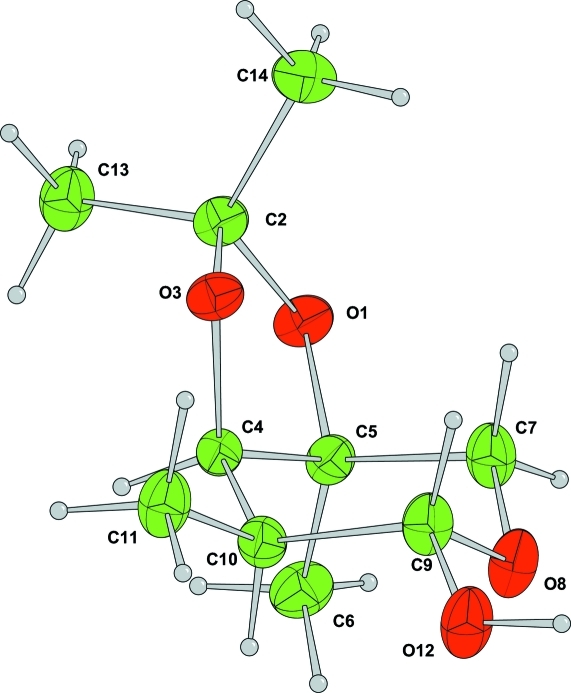
The molecular structure showing the crystallographic labelling scheme. Displacement ellipsoids drawn at the 50% probability level. H atoms are shown as spheres of arbitary radius.

**Fig. 3.**
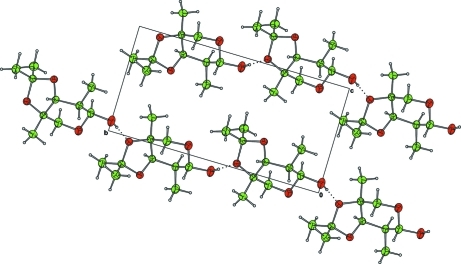
Packing diagram for the title compound projected along the a-axis. Hydrogen bonds are indicated by dotted lines.

## supplementary crystallographic information

### Comment

Deoxy sugars play an important role in the natural world; 2-deoxy ribose forms the sugar backbone of DNA whilst L-fucose, 6-deoxy-L-galactose, is involved in a wide range of mammalian glycan mediated responses (Becker and Lowe, 2003). Whilst the synthesis and biological evaluation of deoxy sugars is relatively common (Yoshihara *et al.*, 2008; Gullapalli *et al.*, 2007), examples of doubly branched analogues are to our knowledge, unknown.

Herein we report the structure of the novel deoxy aldose 3, generated by a short synthetic sequence from di-branched lactone 1 (Booth *et al.* 2007) (Fig. 1). Hydrogenation of the alkene functionality in 2 could give either epimer at position C-2 of lactone 3 or a mixture of both products. The reaction proved to be extremely stereospecific, generating only one product. Direct crystallization of lactone 3 generated poor quality crystals, however, after reduction to the lactol, crystallization was facile and X-ray crystallography showed the product to be the *arabino* compound 4 rather than the *ribo* compound 5. The absolute stereochemistry was determined from the use of 2-*C*-methyl-D-ribono-1,4-lactone as starting material.

The pyranose ring adopts a chair conformation with methyl group at position 2 (atom C10 in the crystallogrphic labelling scheme) in the equatorial position (Fig. 2). The crystal structure exists O—H···O hydrogen-bonded chains of molecules lying parallel to the *b*-axis (Fig. 3). Only classical hydrogen bonding has been considered. There are no unusual crystal packing features.

### Experimental

The title compound was recrystallized from dichloromethane by slow evaporation: m.p. 349–352 K; [α]_D_^25^ -49.6 (*c*,0.15 in CHCl_3_).

### Refinement

In the absence of significant anomalous scattering, Friedel pairs were merged and the absolute configuration was assigned from the starting material.

The H atoms were all located in a difference map, but those attached to carbon atoms were repositioned geometrically. The H atoms were initially refined with soft restraints on the bond lengths and angles to regularize their geometry (C—H in the range 0.93–0.98, O—H = 0.82 Å) and *U*_iso_(H) (in the range 1.2–1.5 times *U*_eq_ of the parent atom), after which the positions were refined with riding constraints.

### Crystal data

**Table:**

C_10_H_18_O_4_	*F*(000) = 220
*M**_r_* = 202.25	*D*_x_ = 1.240 Mg m^−^^3^
Monoclinic, *P*2_1_	Mo *K*α radiation, λ = 0.71073 Å
Hall symbol: P 2yb	Cell parameters from 1185 reflections
*a* = 6.0641 (3) Å	θ = 5–27°
*b* = 13.4016 (7) Å	µ = 0.10 mm^−^^1^
*c* = 6.8287 (3) Å	*T* = 150 K
β = 102.596 (2)°	Plate, colourless
*V* = 541.60 (5) Å^3^	0.50 × 0.20 × 0.20 mm
*Z* = 2	

### Data collection

**Table:**

Nonius KappaCCD diffractometer	1183 reflections with *I* > 2σ(*I*)
graphite	*R*_int_ = 0.036
ω scans	θ_max_ = 27.5°, θ_min_ = 5.5°
Absorption correction: multi-scan (*DENZO*/*SCALEPACK*; Otwinowski & Minor, 1997)	*h* = −7→7
*T*_min_ = 0.89, *T*_max_ = 0.98	*k* = −13→17
5025 measured reflections	*l* = −8→8
1266 independent reflections	

### Refinement

**Table:**

Refinement on *F*^2^	Primary atom site location: structure-invariant direct methods
Least-squares matrix: full	Hydrogen site location: inferred from neighbouring sites
*R*[*F*^2^ > 2σ(*F*^2^)] = 0.030	H-atom parameters constrained
*wR*(*F*^2^) = 0.073	Method = Modified Sheldrick *w* = 1/[σ^2^(*F*^2^) + (0.03*P*)^2^ + 0.12*P*], where *P* = [max(*F*_o_^2^,0) + 2*F*_c_^2^]/3
*S* = 0.98	(Δ/σ)_max_ = 0.0001
1266 reflections	Δρ_max_ = 0.16 e Å^−^^3^
127 parameters	Δρ_min_ = −0.16 e Å^−^^3^
1 restraint	

### Fractional atomic coordinates and isotropic or equivalent isotropic displacement parameters (Å^2^)

**Table:**

	*x*	*y*	*z*	*U*_iso_*/*U*_eq_	
O1	0.3944 (2)	0.39582 (13)	0.04435 (17)	0.0245	
C2	0.4077 (3)	0.39975 (17)	0.2596 (2)	0.0228	
O3	0.3257 (2)	0.30533 (13)	0.30874 (17)	0.0224	
C4	0.1684 (3)	0.27174 (16)	0.1342 (2)	0.0195	
C5	0.2816 (3)	0.30472 (16)	−0.0350 (2)	0.0213	
C6	0.1137 (3)	0.32628 (18)	−0.2291 (3)	0.0299	
C7	0.4647 (3)	0.23026 (16)	−0.0612 (3)	0.0275	
O8	0.3798 (2)	0.13095 (14)	−0.0775 (2)	0.0287	
C9	0.3274 (3)	0.10066 (16)	0.1088 (3)	0.0255	
C10	0.1251 (3)	0.15996 (16)	0.1439 (3)	0.0220	
C11	0.0585 (3)	0.13084 (17)	0.3391 (3)	0.0294	
O12	0.2706 (2)	0.00005 (14)	0.0917 (2)	0.0322	
C13	0.2595 (3)	0.48536 (17)	0.3003 (3)	0.0298	
C14	0.6502 (3)	0.41048 (18)	0.3718 (3)	0.0312	
H41	0.0240	0.3081	0.1185	0.0230*	
H63	0.0358	0.2638	−0.2803	0.0479*	
H62	0.1938	0.3530	−0.3250	0.0474*	
H61	0.0028	0.3745	−0.2037	0.0467*	
H71	0.5148	0.2448	−0.1837	0.0319*	
H72	0.5917	0.2377	0.0580	0.0331*	
H91	0.4639	0.1091	0.2207	0.0329*	
H101	0.0017	0.1448	0.0295	0.0264*	
H111	0.0082	0.0613	0.3332	0.0474*	
H112	0.1891	0.1410	0.4520	0.0465*	
H113	−0.0662	0.1730	0.3609	0.0474*	
H132	0.2647	0.4876	0.4458	0.0489*	
H131	0.3214	0.5481	0.2611	0.0489*	
H133	0.1041	0.4759	0.2247	0.0491*	
H142	0.6552	0.4165	0.5154	0.0457*	
H143	0.7110	0.4711	0.3212	0.0458*	
H141	0.7362	0.3515	0.3460	0.0456*	
H121	0.3746	−0.0319	0.0515	0.0539*	

### Atomic displacement parameters (Å^2^)

**Table:**

	*U*^11^	*U*^22^	*U*^33^	*U*^12^	*U*^13^	*U*^23^
O1	0.0310 (7)	0.0222 (6)	0.0214 (6)	−0.0072 (5)	0.0082 (5)	−0.0002 (5)
C2	0.0259 (8)	0.0224 (9)	0.0208 (7)	−0.0045 (7)	0.0066 (6)	−0.0005 (7)
O3	0.0270 (6)	0.0202 (7)	0.0199 (6)	−0.0045 (5)	0.0047 (5)	−0.0001 (5)
C4	0.0190 (8)	0.0193 (9)	0.0205 (8)	−0.0003 (6)	0.0050 (6)	−0.0018 (6)
C5	0.0233 (8)	0.0199 (9)	0.0215 (8)	−0.0012 (7)	0.0069 (6)	−0.0017 (7)
C6	0.0358 (10)	0.0297 (11)	0.0223 (9)	0.0016 (8)	0.0025 (7)	0.0005 (8)
C7	0.0276 (9)	0.0248 (10)	0.0340 (10)	0.0003 (8)	0.0153 (8)	0.0019 (8)
O8	0.0363 (7)	0.0218 (7)	0.0333 (7)	0.0021 (6)	0.0190 (6)	0.0004 (6)
C9	0.0284 (9)	0.0196 (9)	0.0312 (9)	0.0013 (7)	0.0124 (7)	0.0023 (7)
C10	0.0212 (8)	0.0201 (9)	0.0257 (9)	−0.0015 (6)	0.0074 (6)	−0.0011 (7)
C11	0.0344 (10)	0.0236 (9)	0.0349 (10)	0.0002 (8)	0.0176 (8)	0.0011 (8)
O12	0.0366 (7)	0.0200 (7)	0.0450 (8)	0.0021 (6)	0.0198 (6)	−0.0011 (6)
C13	0.0327 (10)	0.0243 (10)	0.0354 (10)	−0.0004 (8)	0.0141 (8)	−0.0007 (8)
C14	0.0276 (9)	0.0324 (11)	0.0313 (9)	−0.0037 (8)	0.0013 (7)	−0.0021 (9)

### Geometric parameters (Å, °)

**Table:**

O1—C2	1.4553 (19)	O8—C9	1.436 (2)
O1—C5	1.446 (2)	C9—C10	1.524 (2)
C2—O3	1.426 (2)	C9—O12	1.390 (2)
C2—C13	1.520 (3)	C9—H91	1.003
C2—C14	1.510 (2)	C10—C11	1.525 (2)
O3—C4	1.427 (2)	C10—H101	0.978
C4—C5	1.533 (2)	C11—H111	0.979
C4—C10	1.525 (2)	C11—H112	0.987
C4—H41	0.987	C11—H113	0.981
C5—C6	1.513 (2)	O12—H121	0.855
C5—C7	1.532 (2)	C13—H132	0.988
C6—H63	0.986	C13—H131	0.982
C6—H62	0.965	C13—H133	0.979
C6—H61	0.975	C14—H142	0.978
C7—O8	1.423 (2)	C14—H143	0.986
C7—H71	0.970	C14—H141	0.984
C7—H72	0.996		
			
C2—O1—C5	109.06 (12)	C7—O8—C9	109.94 (14)
O1—C2—O3	105.09 (13)	O8—C9—C10	109.46 (14)
O1—C2—C13	107.90 (14)	O8—C9—O12	107.37 (15)
O3—C2—C13	112.10 (14)	C10—C9—O12	109.02 (14)
O1—C2—C14	110.45 (13)	O8—C9—H91	109.8
O3—C2—C14	108.43 (15)	C10—C9—H91	112.3
C13—C2—C14	112.62 (16)	O12—C9—H91	108.7
C2—O3—C4	106.69 (12)	C4—C10—C9	110.70 (13)
O3—C4—C5	102.17 (13)	C4—C10—C11	111.73 (15)
O3—C4—C10	111.32 (14)	C9—C10—C11	112.32 (15)
C5—C4—C10	115.19 (14)	C4—C10—H101	106.2
O3—C4—H41	110.5	C9—C10—H101	105.6
C5—C4—H41	108.0	C11—C10—H101	110.0
C10—C4—H41	109.4	C10—C11—H111	110.3
C4—C5—O1	102.36 (13)	C10—C11—H112	109.1
C4—C5—C6	112.89 (15)	H111—C11—H112	110.6
O1—C5—C6	109.89 (15)	C10—C11—H113	110.2
C4—C5—C7	110.82 (15)	H111—C11—H113	108.2
O1—C5—C7	107.33 (13)	H112—C11—H113	108.4
C6—C5—C7	112.89 (15)	C9—O12—H121	109.0
C5—C6—H63	109.1	C2—C13—H132	108.4
C5—C6—H62	108.8	C2—C13—H131	108.7
H63—C6—H62	110.3	H132—C13—H131	108.6
C5—C6—H61	109.2	C2—C13—H133	110.2
H63—C6—H61	109.3	H132—C13—H133	110.5
H62—C6—H61	110.1	H131—C13—H133	110.4
C5—C7—O8	111.05 (14)	C2—C14—H142	109.3
C5—C7—H71	109.9	C2—C14—H143	107.3
O8—C7—H71	107.2	H142—C14—H143	110.6
C5—C7—H72	106.9	C2—C14—H141	109.1
O8—C7—H72	111.1	H142—C14—H141	110.1
H71—C7—H72	110.7	H143—C14—H141	110.3

### Hydrogen-bond geometry (Å, °)

**Table:**

*D*—H···*A*	*D*—H	H···*A*	*D*···*A*	*D*—H···*A*
C6—H61···O12^i^	0.97	2.59	3.562 (3)	173
O12—H121···O1^ii^	0.86	1.93	2.786 (3)	179

Symmetry codes: (i) −*x*, *y*+1/2, −*z*; (ii) −*x*+1, *y*−1/2, −*z*.
